# Maternal Fructose Exposure Programs Metabolic Syndrome-Associated Bladder Overactivity in Young Adult Offspring

**DOI:** 10.1038/srep34669

**Published:** 2016-10-05

**Authors:** Wei-Chia Lee, You-Lin Tain, Kay L. H. Wu, Steve Leu, Julie Y. H. Chan

**Affiliations:** 1 Division of Urology, Kaohsiung Chang Gung Memorial Hospital and Chang Gung University College of Medicine, Kaohsiung, Taiwan; 2 Department of pediatrics, Kaohsiung Chang Gung Memorial Hospital and Chang Gung University College of Medicine, Kaohsiung, Taiwan; 3 Institute for Translational Research in Biomedicine, Kaohsiung Chang Gung Memorial Hospital, Kaohsiung, Taiwan

## Abstract

Maternal fructose exposure (MFE) programs the development of metabolic syndrome (MetS) in young adult offspring. Epidemiological data indicate that MetS may increase the risks of overactive bladder (OAB) symptoms. However, it remains unknown whether MFE programs MetS-associated bladder dysfunction in adult offspring. Using Sprague-Dawley rats, we investigated the effects of MFE during pregnancy and lactation on developmental programming of MetS-associated bladder dysfunction. In addition, next generation sequencing technology was used to identify potential transcripts involved in the programmed bladder dysfunction in adult male offspring to MFE. We found that MFE programmed the MetS-associated OAB symptoms (i.e., an increase in micturition frequency and a shortened mean inter-contractile interval) in young adult male offspring, alongside significant alterations in bladder transcripts, including *Chrm2, Chrm3, P2rx1, Trpv4,* and *Vipr2* gene expression. At protein level, the expressions of M_2_-, M_3_-muscarinic and P2X_1_ receptor proteins were upregulated in the MFE bladder. Functionally, the carbachol-induced detrusor contractility was reduced in the MFE offspring. These data suggest that alterations in the bladder transcripts and impairment of the bladder cholinergic pathways may underlie the pathophysiology of programmed bladder dysfunction in adult offspring to MFE.

Overactive bladder (OAB) is a cluster of symptoms that include urinary urgency, usually accompanied by frequency and nocturia with or without urge incontinence^1^. Patients with symptoms of OAB may curtail their participation in social activities, affecting their quality of life. Usually, the increasing prevalence of OAB is associated with aging processes. However, even among young male adults, the estimated prevalence of OAB is between 7% and 17% in different ethnic groups^2^. There is increasing evidence linking features of metabolic syndrome (MetS) with OAB^1^,^3^. In several animal models of MetS, bladder overactivity is the major phenotype of voiding behavior associated with metabolic bladder dysfunction^4^,^5^,^6^. Epidemiological studies further suggest that people with features of MetS may have an increased risk of OAB symptoms^3^,^7^.

The prevalence of MetS, a prediabetic condition consisting a group of cardiovascular risk factors including hypertension, dyslipidemia, central obesity, and impaired glucose tolerance^8^, has rapidly grown in parallel with fructose consumption and rapid economic development in Asia^8^,^9^. In the young adult population in China, the estimated prevalence of prediabetes is >40%^10^. According to the concept of the developmental origins of health and disease, parental effects, including heritable genetic, epigenetic, and environmental factors, can affect the subsequent generation’s risk of ill health^11^. It has been suggested that adult MetS can be programmed by intrauterine factors and the early postnatal environment, a phenomenon known as “fetal programming of adult disease”^12^. Pre- and post-natal nutritional insults (e.g., a high-fructose diet) may induce tissue programming and physiological processes of developmental plasticity, leading to MetS and associated comorbidity in adult offspring^13^. It is now recognized that maternal fructose exposure (MFE) during gestation and lactation may alter the development of the fetal metabolic system and induce programmed hypertension^14^ and insulin resistance^15^ in adult offspring.

Whether MFE programs OAB in adult offspring in association with MetS is currently unknown. In addition, mechanism underlying the programmed OAB also remains unclear. We conducted the present study to investigate the effect of MFE during pregnancy and lactation on the development of MetS-associated bladder dysfunctions in young adult male offspring, and performed the next generation sequencing (NGS) analysis to identify potential transcripts associated with the programmed bladder dysfunction in the MFE offspring.

## Results

### General characteristics and voiding behavior

**Figure 1:**
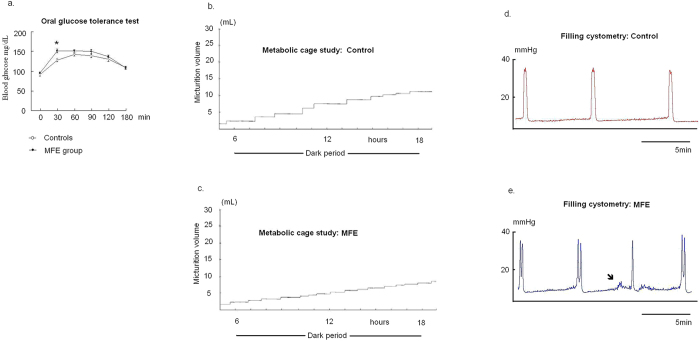
The phenotype of MFE offspring in insulin resistance and voiding behavior. (**a**) OGTT test: the response curve of plasma glucose to OGTT test at the age of 12-week of experimental animals. Points represent mean ± SE of 12 observations. Asterisk indicates significant difference between controls and MFE rats (*t*-test, p = 0.005). (**b**,**c**) Metabolic cage study: a MFE rat showed the increased micturition frequency. (**d**,**e**) Filling cystometry: The shorten ICIs of cystometry characterized bladder overactivity in MFE rats. The non-void contractions were observed in some MFE rats (arrow).

The litter sizes (high fructose diet: 11.6 ± 0.7; control: 11.8 ± 0.8) and sex ratio (male:female ≅ 2:3) were not significantly affected by MFE. Table 1 and Fig. 1 demonstrate the general physical characteristics and biochemical results in both experimental groups. Compared to controls, offspring of the MFE group showed the increases in mean systolic and diastolic pressure, triglycerides, and micturition frequency, along with a shortened mean inter-contractile interval during cystometry. A significant spike of serum glucose level was observed in a 30-min oral glucose tolerance test (OGTT) in the MFE group (*p* < 0.01) (Fig. 1a). In contrast, there was no significant difference in body weight of offspring (Table 1) between the MFE and control animals.

### Bladder function–related gene expression

**Figure 2:**
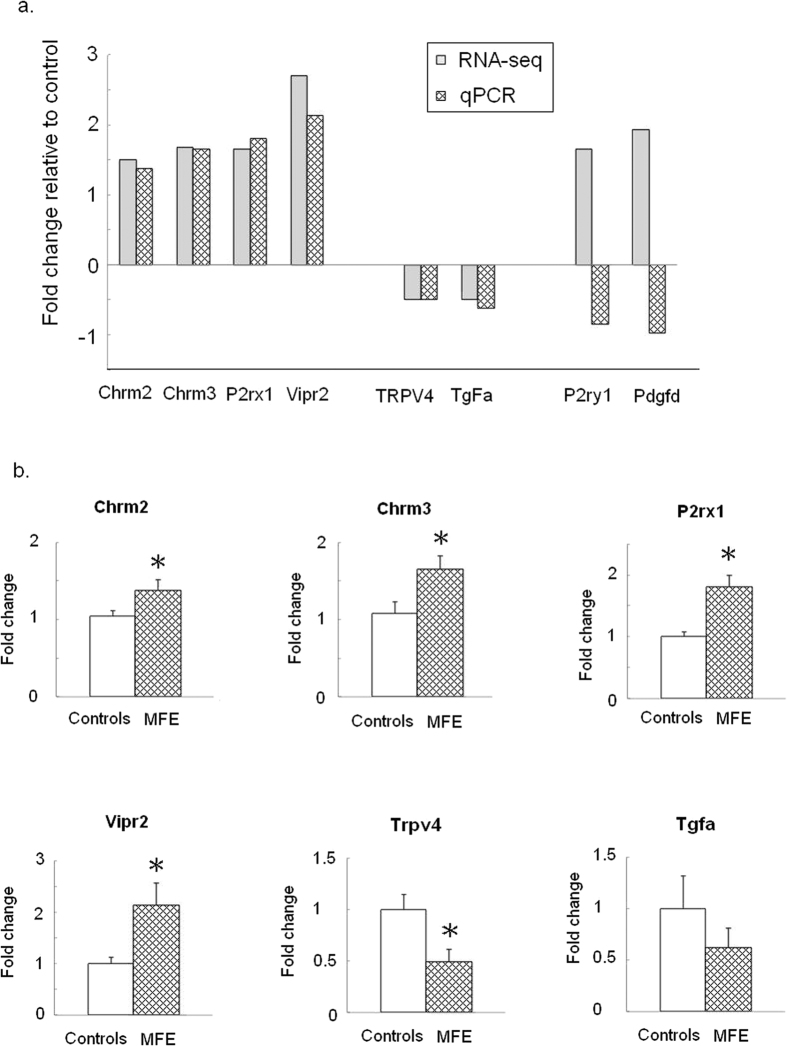
Effect of MFE on gene expression in bladder of the offspring at the age of 12 week. (**a**) Confirmatory analysis of genes derived from RNA-Seq by qPCR in MFE versus control groups. (**b**) Validation of genes expression by qPCR. Data are represented in mean ± SE of 8 observations per group. Asterisk indicates significant difference between controls and MFE rats (p < 0.05).

As shown in Table 2, the gene expressions of *Chrm2, Chrm3, P2rx1, P2ry1, Trpv4, Tgfa, Vipr2,* and *Pdgfd* were found to be modified above the chosen threshold in the bladders of the MFE offspring compared to those of controls. Subsequent quantitative real-time PCR (qPCR) validation revealed significantly increased mRNA levels transcribed from *Chrm2 (p* = 0.04)*, Chrm3 (p* = 0.03)*, P2rx1 (p* < 0.01), and *Vipr2 (p* < 0.01) and significantly decreased levels of mRNA transcribed from *Trpv4 (p* < 0.01) of the bladders in the MFE group (Fig. 2b).

### Assessment of M2- and M3-mAChR and P2X_1_ receptor proteins in the bladder

**Figure 3:**
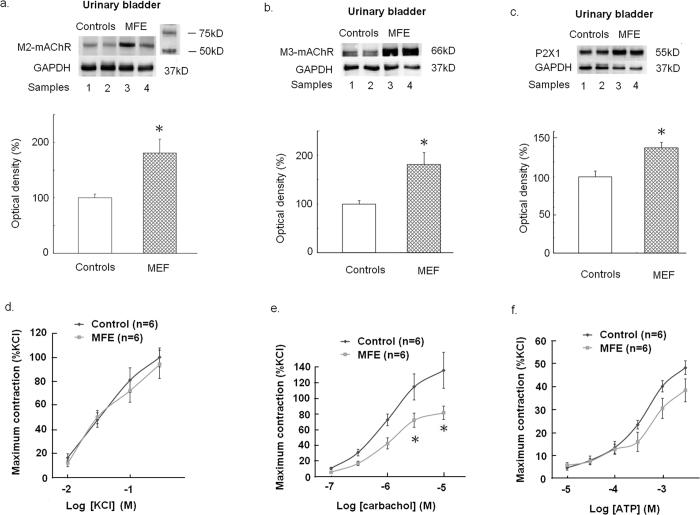
Bladder functional protein and contractility assessments. (**a**–**c**) representative blots and changes in average functional protein expression in the bladder of adult offspring assessed by Western blot analysis with specific antibodies to M2 and M3-mAChRs, purinergic P2X_1_ receptor and GAPDH. Full-length blots/gels are presented in Supplementary Fig. S2. Experiments were repeated two times and representative blots are shown (upper panels). Data of proteins expression (ratios of signal intensities of investigated proteins relative to GAPDH) were calculated with 8 samples in each group. Data which were standardized and expressed in percentage to the control group (lower panels) (**d**–**e**) The concentration-response curves (*n* = 6, in each group) of the denuded detrusor muscle in response to the treatment with different doses of KCl, carbachol or ATP. In each experiment contractile response was expressed as percent of 300 mM KCl-induced mean contractility of the control group. All data are presented in Mean ± SE. An asterisk indicates a significant difference between controls and the MFE group (Two samples *t* test, p < 0.05).

According to the Western blotting for measurement of protein expression, offspring of the MFE group showed significant increases in M2- and M3- muscarinic acetylcholine receptor (mAChR) (*p* = 0.039 and *p* < 0.01), as well as P2X_1_ (*p* = 0.032) protein expression in the bladder (Fig. 3a–c).

### Detrusor contractility

Concentration–response curves of KCl, carbachol, and ATP for detrusor strips from offspring of controls and the MFE group are shown in Fig. 3d–f. The contractile responses to high concentrations of carbachol showed a significant decrease in the MFE offspring (p = 0.021 and p = 0.01). In contrast, the contractile responses to KCl and ATP showed no significant difference between MFE offspring and controls.

## Discussion

Results of this study show that MFE during gestation and lactation may induce bladder overactivity and urinary frequency in young adult male offspring. Among the 20 target gene families involved in bladder physiological functions, we identified significant increases in *Chrm2, Chrm3, P2rx1, Vipr2,* and a significant decrease in *Trpv4* mRNA levels; among them the expressions of M2- and M3-mAChR and P2X_1_ receptor proteins were increased in the bladder of the MFE offspring. In the bladder functional tests, the MFE offspring showed a significant decreased in carbachol-induced detrusor contractility. Together these observations suggest that bladder dysfunction in adult male offspring programmed by MFE is associated with alterations in the bladder transcripts, impairment of the cholinergic pathways of the bladder, and promotion of OAB symptoms.

Our results support the concept of the developmental origins of health and disease, in which pre- and postnatal high fructose exposure induces tissue programming, leading to MetS and associated comorbidity in adult offspring^11^,^12^,^13^,^14^. Mechanisms underlying the developmental programming of bladder overactivity in adult offspring to MFE are not immediate clear. Epigenetic regulation by means of DNA methylation, covalent histone modifications, microRNA changes, and polycomb-group complex recruitment of genes encoding the metabolism-related proteins has been proposed^11^,^12^,^13^,^14^,^16^. In addition, it was reported that chemical insults during the neonatal period of rats induce long-term visceral sensory plasticity in the bladder^17^, prompting the speculation of an early metabolic perturbation acting on the vesical nerve system. This may, in turn, change the neural transmission that could last well into the adulthood for primary or secondary programming of the disease.

Our results demonstrate that the impaired cholinergic contraction system and upregulated expression of M2- and M3-mAChR and P2X_1_ receptors in the bladder are associated with OAB symptoms in adult male offspring to MFE. The OAB symptom is a common phenotype in animals with MetS-associated bladder dysfunction, of which upregulation of M2- and M3-mAChR mRNA and proteins, as well as the decreased cholinergic contraction of the bladder are prominent^4^,^5^,^6^. We found in the present study that the same processes are engaged in the developmental programming of bladder dysfunction, highlighting their significance in the pathophysiology of bladder dysfunction associated with MetS. It is well known that M3-mAChR–mediated detrusor contraction via rho/rho kinase pathways is responsible for the normal micturition contraction^18^. Notably we found in this study a reduction in the carbachol (a mAChR agonist)-induced detrusor contractility despite the upregulated protein expressions of the mAChRs in bladder of MFE offspring. In this regard, activities of Rho-A and protein kinase C have been demonstrated to be decreased during carbachol stimulation in the spontaneous hypertensive rats^19^. In this study we found the MFE offspring showed the characteristic of hypertension (cf. Table 1). We therefore speculate that the decrease in carbachol-induced contractility in MFE offspring might be attributable to the decreased activity of Rho-A and protein kinase C activity despite an increase in the expression of mAChRs protein in the bladder. This speculation, however, awaits further validation. M2-mAChR may counteract β_3_-adrenoceptors by reducing intracellular levels of cyclic adenosine monophosphate to inhibit the relaxation of the bladder^18^. Distributions of M2- and M3-mAChR in the urothelium facilitate afferent activity and initiate micturition^1^. However, pathologic states such as partial denervation or myopathy of the bladder may lead to compensated upregulation of M2- and M3-mAChR, which is one of the casual factors of OAB in humans^18^. Additionally, parasympathetic nerves that supply the urinary bladder use acetylcholine and ATP as cotransmitters to elicit detrusor contraction^1^. Upregulation of P2X_1_ receptors in the bladder may thus have a prominent role in provoking detrusor overactivity^18^.

Our qPCR results also demonstrated a decrease in *Trpv4* gene expression and an increase in *Vipr2* gene expression in the bladder of MFE offspring. The *Trpv4* gene encodes an osmo-sensitive TRPV4 ion channel protein. In the bladder, inhibition of TRPV4 receptors increase functional bladder capacity and reduce micturition frequency^20^. Thus, the decreased expression of TRPV4 could be a part of compensatory processes for reducing the bladder overactivity and possible OAB symptoms in the MFE bladder. The TRPV4 is also involved in the energy homeostasis and glucose-stimulated insulin secretion in the pancrease^21^. The VPAC_2_ receptor is a G-protein–coupled receptor that is encoded by the *Vipr2* gene. VPAC_2_ receptors are widely expressed in urinary bladder^22^. The main endogenous peptide ligands of VPAC_2_ are VIP and pituitary adenylate cyclase–activating polypeptide (PACAP). It was suggested that the VIP/PACAP system could relax the smooth muscle of the lower urinary tract^23^. In the insulin-secreting cells, activation of the VPAC_2_ enhances glucose-induced insulin secretion^24^. Taken together, alterations in TRPV4 and VPAC_2_ receptor mRNA expression in the bladder of the MFE offspring suggest a linkage among nutrient sensing, energy metabolism, and bladder dysfunction.

The developmental programming of OAB is of high relevance in clinic medicine and public health. Our findings that the processes that produce a propensity to MetS and associated bladder dysfunction could be established early in life pose the question of whether the interventions in adults are likely to come too late to be effective in offspring to the MFE.

## Conclusions

Our results demonstrate that MFE during gestation and lactation periods can program bladder overactivity in young adult male offspring in a rat model. Based on data from the NGS analysis, it is likely that alterations in the bladder transcripts, including increased mRNA levels of M2- and M3-mAChR, P2X_1_ receptor, and VPAC_2_ receptor, along with decreased mRNA levels of TRPV4 receptor, may be associated with the primary or secondary programmed bladder dysfunction in the MFE offspring. However, their roles in the impaired detrusor cholinergic contraction in the MFE offspring require further investigation.

## Materials and Methods

### Animals

This study was performed in accordance with the guidelines of National Research Council, USA, and the Animal Protection Law by the Council of Agriculture of the Republic of China. The experimental protocol was approved by the Institutional Animal Ethics Committee of Chang Gung Memorial Hospital (Permit Number 201332703). All surgeries were performed under urethane (1.2 g/kg) anesthesia, and every effort was made to minimize suffering and the number of animals used throughout the experiment.

Virgin Sprague-Dawley rats (12–16 week old, *n* = 8) were obtained (BioLASCO Taiwan Co., Ltd., Taipei, Taiwan) and maintained in a facility accredited by the Association for Assessment and Accreditation of Laboratory Animal Care International under temperature control (24 ± 0.5 °C) and a 12:12-h light:dark cycle. Male Sprague-Dawley rats were caged with individual females until mating was confirmed by observation of a vaginal plug. In some animals this was further validated by the detection of spermatozoa by vaginal lavage. Pregnant Sprague-Dawley rats were randomly assigned to receive regular chow or a fructose-rich diet (60% fructose diet, Harlan Teklad, Madison, WI, USA) during the whole period of pregnancy and lactation. To ensure homogeneity of evaluated offspring, all litters were adjusted to 12 pups per dam. Following weaning male offspring were selected from the litters (controls or MFE rats, *n* = 18 in each group) and were used in subsequent experiments. Among them, 12 rats in each group were subjected to the filling cystometry; eight of them were used for subsequent qPCR and Western analyses and the remaining 4 rats were sent for NGS analyses. The other 6 rats were used for the *in vitro* organ bath experiments. Only male rats were used because they are likely to develop metabolic traits at a higher rate in their late life, whereas the female offspring seems having less impacts in this model^15^,^16^.

### Metabolic cage study and OGTT

At the age of 12 week, 12 rats in each group were placed in individual 3701M081 metabolic cages (Tecniplast, Buguggiate, Italy). After a 24-h familiarization period, a known volume of water was measured and placed in the animals’ drinking bottles. Micturition frequency and urine output were determined using a cup specially fitted to an FT-104 force transducer (iWorx/CB Sciences, Inc., Dover, NH, USA). The volume of the liquid consumed, 24-h micturition frequency, and urine production were then recorded for 3 days by a physiological recorder (iWorx 308; iWorx/CB Sciences, Inc.), and an average value was determined. This was followed by an OGTT which was performed after an overnight fast. Rats were administrated with 1 g/kg glucose by oral gavage, and blood sugar levels were measured before, 30, 60, 90, 120, and 180 min after oral gavage. Blood samples were collected from the tail vein before and at different time points after glucose intake. The blood glucose levels were measured using a Medisense Precision Q.I.D. glucose meter (Abbott Laboratories, Bedford, MA, USA).

### Plasma biochemical analysis

Blood samples were collected at the end of experiments by a punch at the tip of the tail. The blood was centrifuged at 1000× g for 15 minutes to separate the serum. Serum glucose level was determined by the glucose oxidase method (Roche, Basel, Switzerland). The concentration of triglycerides was analyzed by triglycerides assay kit (Randox, Antrim, UK), and total cholesterol was analyzed by means of colorimetric enzymatic methods (Sigma-Aldrich, St Louis, MO, USA).

### Filling Cystometry

The same twelve rats in each group were weighed and then anesthetized with subcutaneous urethane (1.2 g/kg). Polyethylene-50 catheters were placed in the left carotid artery to measure arterial pressure using a PowerLab 16S system with a P23 1D transducer (Gould-Statham, Oxnard, CA, USA). Via the suprapubic approach, the bladder catheter was connected via a T tube to a pressure transducer and a microinjection pump (CH-4103; Infors, Bottmingen, Switzerland). Room-temperature saline was infused into the bladder at a rate of 0.08 mL/min. Voiding pressure was recorded on an RS3400 chart recorder (Gould, Cleveland, OH, USA). All rats were allowed for a minimum of 30 min baseline recording in order for the voiding pattern to stabilize. Thereafter, reproducible micturition cycles were recorded for 1-h periods and were used for evaluation. An overdose of urethane was then injected to sacrifice the rats. Blood samples were collected for biochemical analysis.

### NGS and data analysis

Urinary bladders were isolated and snap frozen for whole-genome RNA NGS (RNA-Seq) performed by Welgene Biotech Co., Ltd. (Taipei, Taiwan). Purified RNA was quantified at 260 nm (OD_600_) using an ND-1000 spectrophotometer (Nanodrop Technology, Wilmington, DE, USA) and was analyzed using a Bioanalyzer 2100 (Agilent Technologies Inc., Santa Clara, CA, USA) with an RNA 6000 LabChip kit (Agilent Technologies Inc.) All procedures were performed according to the Illumina (San Diego, CA, USA) protocol. For all samples, library construction was performed using the TruSeq RNA Sample Prep Kit v2 for approximately 160-bp (single-end) sequencing and the Solexa platform. The reference genome and gene annotations were retrieved from Ensembl database (http://asia.ensembl.org/index.html). The sequence was directly determined by sequencing-by-synthesis technology using the TruSeq SBS Kit. Raw sequences were obtained using the Illumina GA Pipeline software CASAVA v1.8, which was expected to generate 30 million reads per sample. These raw sequences then went through a filtering process to obtain qualified reads. ConDeTri was implemented to trim or remove the reads according to the quality score. Numbers of reads after quality trim ranged between 31∼39 millions. Qualified reads after filtering low-quality data were analyzed using TopHat/Cufflinks for gene expression estimation. The gene expression level was calculated as fragments per kilobase of transcript per million mapped reads^14^. The open-source Cuffdiff tool from the Cufflinks package was run to calculate expression changes and associated *q* values (false discovery rate–adjusted *p* values) for each gene between control and MFE rats. The Cuffdiff output files were further annotated by adding gene functional descriptions and Gene Ontology classifications^14^,^16^. The reference genome and gene annotations were retrieved from the Ensembl database. Following a literature review^25^, genes of 20 ionic channels/receptor families that are crucial for bladder function and are expressed within the bladder were screened.

### RNA extraction and qPCR analysis

Isolation of RNA was performed using TRIzol Reagent (Life Technologies, Grand Island, NY, USA) according to the manufacturer’s instructions: 1 mg of total RNA was taken and reversely transcribed using the iScript Reverse Transcription Supermix (Bio-Rad, Hercules, CA, USA), and the resulting cDNA was used as the PCR template. The mRNA levels were determined by real-time PCR with the CFX Connect qPCR Detection System (Bio-Rad). GAPDH was used as an endogenous control. The PCR reaction mixture contained the KAPA SYBR FAST qPCR Master Mix (2x) Universal kit (Kapa Biosystems, Wilmington, MA, USA), cDNA, and the primers. The genes validated by qPCR included mAChR M2 and M3 (*Chrm2* and *Chrm3*), P2X purinoceptor 1 (*P2rx1*), P2Y purinoceptor 1 (*P2ry1*), transient receptor potential cation channel subfamily V member 4 (*Trpv4*), VIP receptor 2 (*Vipr2*), transforming growth factor alpha (*Tgfa*), and platelet-derived growth factor D (*Pdgfd*). Rat primers used for qPCR were list in the Supplementary Table S1. All samples were run in triplicate. For the relative quantification of gene expression, the comparative threshold cycle (C_t_) method was employed. The averaged C_t_ was subtracted from the corresponding averaged *R18s* value for each sample, resulting in ΔC_t_. ΔΔC_t_ was achieved by subtracting the average control ΔC_t_ value from the average experimental ΔC_t_. The fold-increase was established by calculating 2^−^ΔΔ^Ct^ for MFE vs. control bladder samples^14^,^16^.

### Western immunoblotting

The whole-bladder samples were obtained, and the expression levels of M2- and M3-mAChR and the P2X_1_ receptor were evaluated by Western immunoblotting as previously described^26^. In brief, frozen tissues of bladder from each group were homogenized on ice in the CelLytic^TM^ MT cell lysis buffer (Sigma-Aldrich) containing the Protease Inhibitor (100 μl pepstain and 26.5 μl aprotinin). Protein concentration in the supernatant was determined using the Pierce 660 nm protein assay (Thermo, Waltham, MA, USA) against a protein standard (Sigma-Aldrich). An equal amount of protein (30 μg) from the bladders was loaded on sodium dodecyl sulfate polyacrylamide electrophoresis gels and transferred to Immobilon-P membranes (Millipore, Billerica, MA, USA). Immobilon-P membranes were then blocked and were then incubated with the primary antibody. Antibodies raised against M2-mAChR (1:1000 dilution; Alomone Labs, Jerusalem, Israel), M3-mAChR (1:1000 dilution; Alomone Labs), P2X_1_ receptor (1:10,000 dilution; Alomone Labs), and GAPDH (1:10,000 dilution; Millipore, Billerica, MA, USA) were used. Membranes were then incubated with appropriate horseradish peroxidase–conjugated secondary antibody. Specific antibody-antigen complex was detected using an enhanced chemiluminescence Western Blot detection system (Thermo fisher Bioscience). The amounts of detected proteins were quantified by ImageJ software (NIH, MD, USA).

### Detrusor contractility

In a separate series of experiments (*n* = 6), the *in vitro* contractility studies were performed as previously described^27^. Two 10 × 2 mm denuded longitudinal strips were prepared from the dorsal part of the bladder body. Each was connected to a force displacement transducer and was placed in the Compact Organ bath system (Panlab, SLU, Barcelona, Spain), in each chamber contained 10 mL physiological saline solution. The solution was aerated with 95% O_2_–5% CO_2_ and was maintained at 37 °C (pH 7.4). Resting tension on the tissues was controlled at 1 g. Concentration–response curves to KCl (10–300 mM), carbachol (0.1–10 μM), or ATP (10 μM to 3 mM) were obtained in a stepwise manner after the response to the previous concentration had attained a plateau.

### Statistical analysis

All data are presented as the mean ± SE. Between group comparisons were performed using an independent 2-sample *t* test. Differences were considered significant at *p* < 0.05.

## Additional Information

**How to cite this article**: Lee, W.-C. *et al.* Maternal Fructose Exposure Programs Metabolic Syndrome-Associated Bladder Overactivity in Young Adult Offspring. *Sci. Rep.* **6**, 34669; doi: 10.1038/srep34669 (2016).

## Supplementary Material

Supplementary Information

## Figures and Tables

**Table 1:** General characteristics, biochemistry and voiding behavior in control and MFE groups (*n* = 12).

	Controls	MFE group	p value
General Characteristics			
Body weight (g)	406.1 ± 13.8	431.3 ± 8.9	0.1
Bladder weight (mg)	112.5 ± 7.2	108.7 ± 8.7	0.74
Systolic blood pressure (mmHg)	117.4 ± 2.1	135.9 ± 4.8	0.003*
Diastolic blood pressure (mmHg)	89.5 ± 2.7	113.1 ± 6.3	0.004*
Fasting biochemistry:			
Triglyceride (mg/dl)	39.9 ± 3.9	99.6 ± 6.2	<0.001*
Cholesterol (mg/dl)	69.0 ± 3.8	76.4 ± 2.9	0.14
Fasting glucose (mg/dl)	91.6 ± 4.1	95.7 ± 2.4	0.41
Metabolic cage study/24 hrs			
No. Voids	24.6 ± 1.3	30.1 ± 1.7	0.038*
Urine output (ml)	26.1 ± 2.2	25.1 ± 1.8	0.72
Water intake (ml)	35.1 ± 3.4	38.9 ± 2.5	0.37
Cystometric parameters			
Voiding pressure (mmHg)	24.1 ± 1.2	26.5 ± 1.1	1.33
Inter-contractile interval (min)	10.4 ± 0.35	7.9 ± 0.44	0.01*

^*^Two sample *t*-test, p < 0.05.

**Table 2:** Changes in genes expression involved in bladder function between the MFE group and controls in the screening of next-generation sequencing.

Gene symbol	Description	MFE	Control	Fold changes MFE/control
Muscarinic acetylcholine receptors				
*Chrm2*	Muscarinic acetylcholine receptor M2	31.689	21.066	1.50
*Chrm3*	Muscarinic acetylcholine receptor M3	6.039	3.608	1.67
P2X purinoceptors				
*P2rx1*	P2X purinoceptor 1	296.761	179.252	1.66
P2Y purinoceptors				
*P2ry1*	P2Y purinoceptor 1	7.787	4.726	1.65
TRP Channels				
*Trpv4*	Transient receptor potential cation channel subfamily V member 4	2.949	5.949	0.49
Transforming growth factor				
*Tgfa*	Transforming growth factor alpha	2.795	5.661	0.49
Vasoactive intestinal peptide				
*Vipr2*	Vasoactive intestinal polypeptide receptor 2	1.166	0.431	2.71
Platelet-derived growth factor				
*pdgfd*	Platelet-derived growth factor D	18.438	9.539	1.93
