# Supplementary material for: Maternal Fructose Exposure Programs Metabolic Syndrome-Associated Bladder Overactivity in Young Adult Offspring
Source: Sci Rep. 2016 Oct 5;6:34669. doi: 10.1038/srep34669 (PMC5050417; doi:10.1038/srep34669)

Maternal Fructose Exposure Programs Metabolic Syndrome-Associated Bladder Overactivity in Young Adult Offspring

Wei-Chia Lee, M.D. Ph.D.[1], You-Lin Tain, M.D. Ph.D.[2,3], Kay L.H. Wu, Ph.D.[3], Steve Leu, Ph.D.[3], Julie Y.H. Chan. Ph.D.[3]*

[1]Division of Urology, Kaohsiung Chang Gung Memorial Hospital and Chang Gung University College of Medicine, Kaohsiung; [2]Department of pediatrics, Kaohsiung Chang Gung Memorial Hospital and Chang Gung University College of Medicine, Kaohsiung, Taiwan; [3] Institute for Translational Research in Biomedicine, Kaohsiung Chang Gung Memorial Hospital, Kaohsiung, Taiwan

***Address correspondence and reprint requests to**: Julie Y.H. Chan. Ph.D., Institute for Translational Research in Biomedicine, Kaohsiung Chang Gung Memorial Hospital, 123 Ta Pei Rd., Kaohsiung City 83301,Taiwan, Republic of China, e-mail: jchan@adm.cgmh.org.tw, Tel. 886 7-7338415;

Fax: 886-7-7338415.

Supplementary Table S1

Oligonucleotide sequence details of all primers

**Chrm2**	
Forward	5'-AGCCCGCAAAATCGTGAA-3'
Reverse	5'-GACATTGTATGGCGCCCAC-3'
**Chrm3**	
Forward	5'-ACCAACTCCTCGGCAGACAA-3'
Reverse	5'-GCGACATCCTCTTCCGCTT-3'
**P2rx1**	
Forward	5'-ACTCAAGGCCATTGTGCAG-3'
Reverse	5'-CACAGTTGCCTGTGCGAAT-3'
**Vipr2**	
Forward	5'-CCGAGGATGAGAGTAAGATCACG-3'
Reverse	5'-AGATGGCTCTCAGCATGAAGG-3'
**Trpv4**	
Forward	5'-GAAGTGTCCGGTGCTGGAGAT-3'
Reverse	5'-ACTTGTCCCTCAGCAGTTCG-3'
**Tgfa**	
Forward	5'-GCAGTGGTGTCTCACTTCAA -3'
Reverse	5'-CACTGCCAGGAGATCTGCATGCTC-3'
**P2ry1**	
Forward	5'- TGACGGTGTTTGCTGTGTCT-3'
Reverse	5'- GGTGGCATAAACCCTGTCGT-3'
**Pdgfd**	
Forward	5'- CAGAGCGCATCCATCAAAGC-3'
Reverse	5'- GCCTGTCACCCGAATGTTCT -3'

Supplementary Figure S2

Full-length blots/gels of bladder functional proteins

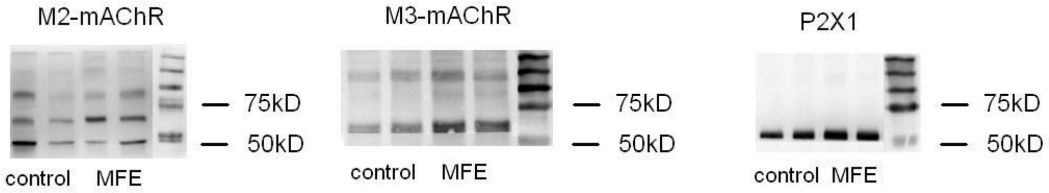

Supplement: Supplementary Information [file srep34669-s1.pdf]
